# Estimating the Number of Molecules in Molecular Junctions Merely Based on the Low Bias Tunneling Conductance at Variable Temperature

**DOI:** 10.3390/ijms232314985

**Published:** 2022-11-29

**Authors:** Ioan Bâldea

**Affiliations:** Theoretical Chemistry, Heidelberg University, Im Neuenheimer Feld 229, D-69120 Heidelberg, Germany; ioan.baldea@pci.uni-heidelberg.de

**Keywords:** molecular electronics, charge nanotransport, electron tunneling, molecular junctions, self-assembled monolayers (SAM), thermal effects, Arrhenius-Sommerfeld thermal transition, area correction factor for large area molecular junctions

## Abstract

Temperature (*T*) dependent conductance G=G(T) data measured in molecular junctions are routinely taken as evidence for a two-step hopping mechanism. The present paper emphasizes that this is not necessarily the case. A curve of lnG versus 1/T decreasing almost linearly (Arrhenius-like regime) and eventually switching to a nearly horizontal plateau (Sommerfeld regime), or possessing a slope gradually decreasing with increasing 1/T is fully compatible with a single-step tunneling mechanism. The results for the dependence of *G* on *T* presented include both analytical exact and accurate approximate formulas and numerical simulations. These theoretical results are general, also in the sense that they are not limited, e.g., to the (single molecule electromigrated (SET) or large area EGaIn) fabrication platforms, which are chosen for exemplification merely in view of the available experimental data needed for analysis. To be specific, we examine in detail transport measurements for molecular junctions based on ferrocene (Fc). As a particularly important finding, we show how the present analytic formulas for G=G(T) can be utilized to compute the ratio $f=A_{\mathrm{eff}}/A_{n}$ between the effective and nominal areas of large area Fc-based junctions with an EGaIn top electrode. Our estimate of $f\approx 0.6\times 10^{-4}$ is comparable with previously reported values based on completely different methods for related large area molecular junctions.

## 1. Introduction

Comparing charge transport properties of single molecule junctions with junctions based on ensembles of molecules represents an important issue that has been frequently addressed in the past [1,2,3,4,5,6]. The former includes mechanically controllable break junctions (MCBJ) [7,8] and scanning tunneling microscopy (STM) break junctions [9,10,11,12] as well as electromigration [13,14,15] platforms. Conducting probe atomic force microscopy (CP-AFM) [16,17,18,19,20], cross-wires [21,22,23,24,25] and large area liquid metal (eutectic gallium indium alloy EGaIn) based molecular junctions [15,26,27,28] are examples of the second category. For the latter, the key role played by the number of molecules and the related effective contact area has been thoroughly emphasized in the literature [5,26,29]. To exemplify, let us refer to the variation of the low bias conductance *G*—the property (determined experimentally from the slope of *I*-*V* curve at low biases where the curve is linear) which will be in the main focus below—across a homologous molecular family whose members contain a variable number of repeat units *n*. Claiming the ubiquitous exponential decay $G_{n}=G_{C}\exp\left(-\tilde{\beta }n\right)$ [10,16,17,20,30,31,32] by monitoring values of conductance $G_{n}$ measured for junctions with various repeat units *n* makes sense only if they contain the same number of molecules. In the same vein, we can mention the tiny even-odd effect reported in the tunneling decay coefficient $\tilde{\beta }$ and/or contact conductance $G_{C}$ [33,34,35,36,37,38,39]. The opposite claims on the direction of this tiny effect (odd members more conductive [27,38] versus even members more conductive [33]) may reflect the difficulty of controlling the effective (“electric”) number of molecules in large area junctions [5,6,26,40,41,42,43,44].

Comparison between temperature dependent transport properties of junctions based on a CP-AFM platform [5] or single-molecule transistors (SET) [15] and large area junctions fabricated with EGaIn technique using the same or similar molecular species has been attempted in the past to address the issue of effective, “electric” area versus nominal, “geometric” area. Nonetheless, the inherently different nature of the contacts of EGaIn- and, e.g., CP-AFM-based junctions (EGaIn top electrode versus AFM metal coated tip) raises some difficulty in interpreting the results of this certainly meaningful approach.

As elaborated below, the approach presented in this paper allows this difficulty to be obviated. It is merely based on low bias conductance data collected on large area junctions at variable temperature. The exact formula for the temperature dependent conductance in the low bias limit deduced recently by us [45] constitutes the theoretical framework of this methodology, which is considered in the next section.

## 2. Results and Discussion

### 2.1. General Results

According to the general Keldysh formalism [46,47,48,49], the low bias conductance $G\equiv {\left.\partial I(V)\partial V\right|}_{V\to 0}$ of a single molecule tunneling junction at finite temperature $\beta ={\left(k_{B}T\right)}^{-1}$ can be expressed as [45,50]

(1) $$\frac{G}{G_{0}}=-\Gamma_{g}^{2}\int_{-\infty }^{\infty }\frac{d\;\varepsilon }{{\left(\varepsilon -\varepsilon_{0}\right)}^{2}+\Gamma_{a}^{2}}\frac{\partial }{\partial \varepsilon }f\left(\varepsilon \right)=\frac{\beta }{4}\Gamma_{g}^{2}\int_{-\infty }^{\infty }\frac{{sech}^{2}\left(\beta \varepsilon /2\right)}{{\left(\varepsilon -\varepsilon_{0}\right)}^{2}+\Gamma_{a}^{2}}d\;\varepsilon$$

Here $G_{0}\equiv 2e^{2}/h=77.48\;\mu$S and $f\left(\varepsilon \right)=1/\left(1+e^{\beta \varepsilon }\right)$ are the quantum conductance and Fermi distribution, respectively, and energies are measured relative to electrodes’ Fermi energy ($E_{F}\equiv 0$). In the present model, the charge transport is mediated by a single level (molecular orbital, MO), and the coupling to two infinite wide, flat band *s* (substrate) and *t* (top, tip) electrodes is quantified by an energy independent effective MO-electrode coupling $\Gamma_{g}$ [51],

(2) $$\Gamma_{g}\equiv \sqrt{\Gamma_{s}\Gamma_{t}}$$

which is the geometric average of the individual MO-electrode couplings $\Gamma_{s,t}$. Effects due to charge image [52,53,54,55,56], gate potential [13], etc that are responsible for level energy shifts are embodied in the renormalized value of $\varepsilon_{0}$, which is a model parameter. In contrast to the isolated molecule, the embedded molecule has an MO possessing a finite energy width

(3) $$\Gamma_{a}\equiv \frac{1}{2}\left(\Gamma_{s}+\Gamma_{t}\right)$$

Equation (1) clearly emphasizes the two distinct impacts of $\Gamma_{s,t}$ on the tunneling transport. On one hand, they contribute multiplicatively via $\Gamma_{g}$ (cf. Equation (2)) as MO-electrode couplings that determine the overall magnitude of the tunneling current. On the other hand, they contribute additively via $\Gamma_{a}$ (Equation (3)) to the MO energy broadening, which can compete with the smearing of the electrodes’ Fermi distributions at nonvanishing temperatures.

As shown recently [45], the RHS of Equation (1) can be integrated out analytically. The result for the conductance per molecule expressed via the real part of Euler’s trigamma function of complex argument function $\psi^{\prime }\left(z\right)$ [57] reads

(4) $$\frac{G}{G_{0}}=\frac{\Gamma_{g}^{2}}{2\pi \Gamma_{a}k_{B}T}Re\;\psi^{\prime }\left(\frac{1}{2}+\frac{\Gamma_{a}}{2\pi k_{B}T}+i\;\frac{\varepsilon_{0}}{2\pi k_{B}T}\right)$$

The trigamma function represents the derivative of the digamma function, $\psi^{\prime }\left(z\right)\equiv \psi \left(1;z\right)\equiv \frac{d}{d\;z}\psi \left(z\right)$, which in turn is the logarithmic derivative of Euler’s gamma function [57]. Equation (4) is an exact result valid at arbitrary values of all parameters ($\varepsilon_{0}$, $\Gamma_{g}$, $\Gamma_{a}$, and *T*).

Noteworthily, *G* does not depend on the sign of $\varepsilon_{0}$. The RHS of Equation (1) is invariant upon changing $\varepsilon_{0}\to -\varepsilon_{0}$. This can easily be seen by changing the variable (ε→−ε). Alternatively, this is also the consequence of the invariance of Equation (4) under complex conjugation. Rephrased physically, junctions wherein conduction is mediated by LUMO ($\varepsilon_{l}=\varepsilon_{0}>0$) and junctions wherein conduction is mediated by HOMO ($\varepsilon_{h}=-\varepsilon_{0}<0$) have the same conductance *G*.

Using the analytic expression Imψ(1/2+iy)=(π/2)tanh(πy) [57], the lowest order Taylor expansion of the RHS of Equation (4) yields

(5) $$\begin{array}{ccc} Re\;\psi^{\prime }\left(\frac{1}{2}+x+iy\right) & = & \frac{\pi^{2}}{2}{sech}^{2}\;\left(\pi y\right)+x\;Re\;\psi \left(2;\frac{1}{2}+iy\right)\\ & + & x^{2}\frac{\pi^{4}}{2}\left[2-\cosh(2\pi y)\right]{sech}^{4}\left(\pi y\right)+O\left(x^{3}\right) \end{array}$$

**Figure 1:**
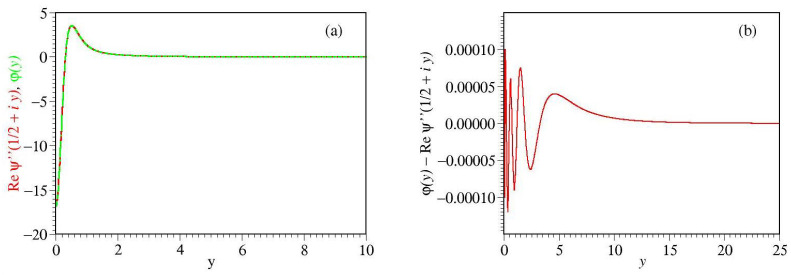
(**a**) The function *φ*(*y*) expressed in terms of elementary functions (Equation (6b)) and the real part of the polygamma function Re *ψ*(2; 1/2 + *iy*) depicted (**b**) along with their differences, revealing that Equation (6) is a very accurate approximation.

The real part of the tetragamma function $\psi \left(2;z\right)\equiv \frac{d^{2}}{dz^{2}}\psi \left(z\right)$ with z=1/2+iy (real *y*) entering above in the RHS is not available in closed analytic form; however, we found that it can be very accurately approximated (Figure 1) via elementary functions as follows [45]


(6a) $$\begin{array}{ccc} & & Re\;\psi \left(2;\frac{1}{2}+iy\right)≃\varphi \left(y\right) \end{array}$$

(6b) $$\begin{array}{ccc} & & \varphi \left(y\right)=\frac{y^{2}-34.7298}{{\left(y^{2}+2.64796\right)}^{2}}+37.262\frac{y^{2}+1.12874}{{\left(y^{2}+2.17786\right)}^{3}}+3.01373\frac{y^{2}-0.082815}{{\left(y^{2}+0.25014\right)}^{3}} \end{array}$$

**Figure 2:**
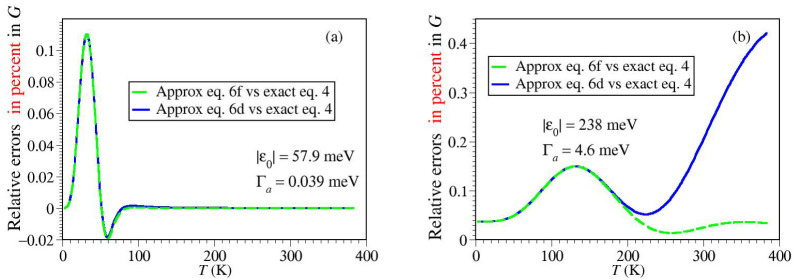
Relative errors in conductance G for parameters (indicated in the legend) characterizing (**a**) the SET and (**b**) SAM setups (see below) illustrating that Equation (6d,f) represent very accurate approximation of the exact Equation (4).

For parameter ranges covering virtually all experimental situations of interest wherein a *T*-dependent *G* can be expected, the parameter

(6c) $$x\equiv \frac{\Gamma_{a}}{2\pi k_{B}T}$$

is small, and the lowest order expansion of the RHS of Equation (4)

(6d) $$\begin{array}{ccc} \frac{G}{G_{0}} & ≃ & \frac{\pi \Gamma_{g}^{2}}{4\Gamma_{a}k_{B}T}{sech}^{2}\pi y+\frac{\Gamma_{g}^{2}\varphi \left(y\right)}{{\left(2\pi k_{B}T\right)}^{2}} \end{array}$$

(6e) $$\begin{array}{ccc} y & \equiv & \frac{\varepsilon_{0}}{2\pi k_{B}T} \end{array}$$

is a very accurate approximation of the exact Equation (4); it holds $O\left(x^{2}\right)$, which amounts to an relative error of ∼1% for $\Gamma_{a}$ smaller than about $k_{B}T/2$. Notice the numerical factor 4 in the denominator of the first term of Equation (6d), which corrects the incorrect factor 16 (a typo) in Equation (6) of ref. [45]. If (highly unlikely in real junctions exhibiting *T*-dependent transport) *x* is not very small with respect to unity, the last term in the RHS of Equation (5) can also be included

(6f) $$\begin{array}{ccc} \frac{G}{G_{0}} & ≃ & \frac{\pi \Gamma_{g}^{2}}{4\Gamma_{a}k_{B}T}{sech}^{2}\pi y+\frac{\Gamma_{g}^{2}\varphi \left(y\right)}{{\left(2\pi k_{B}T\right)}^{2}}\\ & & +\frac{\pi }{16}\frac{\Gamma_{g}^{2}\Gamma_{a}}{{\left(k_{B}T\right)}^{3}}\left[2-\cosh(2\pi y)\right]{sech}^{4}\left(\pi y\right); \end{array}$$

it holds $O\left(x^{3}\right)$, which amounts to an relative error of ∼1% for $\Gamma_{a}$ smaller than about $1.4\;k_{B}T$. At temperatures lower than the aforementioned ($1.4\;k_{B}T\;\overset{<}{∼}\;\Gamma_{a}$), thermal effects are negligible and the zero temperature limit (Equation (8)) applies. Figure 2 illustrates the accuracy of the approximate Equation (6d,f) for parameter values characterizing the real molecular junctions considered in Section 2.3. The curves computed via Equation (6d,f) cannot be distinguished within the drawing accuracy from those obtained via the exact Equation (4) in Section 2.2 and Section 2.3. Therefore, they will not be shown there.

Noteworthily, Equation (6d,f) only contain elementary functions. This is important for practical data fitting; special functions like trigamma entering Equation (4) are usually not implemented in common software packages used by experimentalists.

For parameter values where the peaks of the transmission function and the derivative of the Fermi function—possessing widths of the order $\Gamma_{a}$ and $k_{B}T$, and located at $\varepsilon =\varepsilon_{0}$ and ε=0, respectively—are sufficiently well separated in energy, the following approximate formula

(7) $$\frac{G}{G_{0}}≃\underset{G_{T}/G_{0}}{\underset{︸}{\frac{\pi }{4}\frac{\Gamma_{g}^{2}}{\Gamma_{a}k_{B}T}{sech}^{2}\frac{\varepsilon_{0}}{2k_{B}T}}}+\underset{G_{0K}/G_{0}}{\underset{︸}{\frac{\Gamma_{g}^{2}}{\varepsilon_{0}^{2}+\Gamma_{a}^{2}}}}\overset{\Gamma_{a}\ll \left|\varepsilon_{0}\right|}{⟶}\frac{\pi }{4}\frac{\Gamma_{g}^{2}}{\Gamma_{a}k_{B}T}{sech}^{2}\frac{\varepsilon_{0}}{2k_{B}T}+\frac{\Gamma_{g}^{2}}{\varepsilon_{0}^{2}}$$

generalizes a result deduced earlier [50] for $\Gamma_{s}=\Gamma_{t}=\Gamma_{a}=\Gamma_{g}$. It holds within ∼1% for $\Gamma_{a}$ smaller than about $k_{B}T/10$. Equation (7) reduces in turn to Equations (8) and (9) in the limit of very low and very high temperatures, generalizing results known from earlier studies [15,49,58,59,60].

(8) $$\frac{G}{G_{0}}\overset{k_{B}T\ll \Gamma_{a}}{⟶}\frac{G_{0K}}{G_{0}}=\frac{\Gamma_{g}^{2}}{\varepsilon_{0}^{2}+\Gamma_{a}^{2}}\overset{\Gamma_{a}\ll \left|\varepsilon_{0}\right|}{⟶}\frac{\Gamma_{g}^{2}}{\varepsilon_{0}^{2}}$$

(9a) $$\frac{G}{G_{0}}\overset{\Gamma_{a}\ll \pi k_{B}T}{⟶}\frac{G_{T}}{G_{0}}\equiv \frac{\pi }{4}\frac{\Gamma_{g}^{2}}{\Gamma_{a}k_{B}T}{sech}^{2}\frac{\varepsilon_{0}}{2k_{B}T}$$

(9b) $$\frac{G}{G_{0}}\overset{\Gamma_{a}\ll \pi k_{B}T\ll \left|\varepsilon_{0}\right|}{⟶}\frac{G_{p.A}}{G_{0}}=\frac{\pi \Gamma_{g}^{2}}{\Gamma_{a}k_{B}T}\exp\left(-\frac{\left|\varepsilon_{0}\right|}{k_{B}T}\right)$$

(The above subscript p.A stands for pseudo-Arrhenius).

Notice that unlike Equation (7), *T* enters the RHS of Equation (6d,f) not only in the first term but also in the second term. Therefore, departures of Equation (7) from Equation (4) become substantial when $|\varepsilon_{0}|$, $\Gamma_{a}$, and $k_{B}T$ have comparable values. For this reason, for temperatures around $T_{c}$ (see Equation (11a) below), Equation (4) better quantifies the gradual transition between an Arrhenius-type (high *T*) and a Sommerfeld (low *T*) regime [45] than Equation (7).

Thermal corrections to Equation (8) can alternatively obtained via Sommerfeld expansion of Equation (1) and expressed in terms of the Riemann ζ function [52,61,62]

$$\frac{G}{G_{0}}=\frac{1}{4}\sum_{n=0}^{\infty }\frac{{\left(k_{B}T\right)}^{2n}}{(2n)!}{\left.\frac{\partial^{2n}}{\partial \varepsilon^{2n}}T\left(\varepsilon \right)\right|}_{\varepsilon =0}\int_{-\infty }^{\infty }x^{2n}{sech}^{2}\frac{x}{2}d\;x=\frac{G_{0K}}{G_{0}}+\sum_{n=1}^{\infty }{\left(k_{B}T\right)}^{2n}\left(2-\frac{1}{2^{2(n-1)}}\right)\zeta \left(2n\right)$$

which gives the first Sommerfeld correction (S1, $O\left(T^{2}\right)$)

(10a) $$\frac{G}{G_{0}}≃\frac{\Gamma_{g}^{2}}{\varepsilon_{0}^{2}+\Gamma_{a}^{2}}\left[1+{\left(\pi k_{B}T\right)}^{2}\frac{\varepsilon_{0}^{2}-\Gamma_{a}^{2}/3}{{\left(\varepsilon_{0}^{2}+\Gamma_{a}^{2}\right)}^{2}}\right]$$

and the second Sommerfeld correction (S2, $O\left(T^{4}\right)$)

(10b) $$\frac{G}{G_{0}}≃\frac{\Gamma_{g}^{2}}{\varepsilon_{0}^{2}+\Gamma_{a}^{2}}\left[1+{\left(\pi k_{B}T\right)}^{2}\frac{\varepsilon_{0}^{2}-\Gamma_{a}^{2}/3}{{\left(\varepsilon_{0}^{2}+\Gamma_{a}^{2}\right)}^{2}}+{\left(\pi k_{B}T\right)}^{4}\frac{7}{15}\frac{5\varepsilon_{0}^{4}-10\varepsilon_{0}^{2}\Gamma_{a}^{2}+\Gamma_{a}^{4}}{{\left(\varepsilon_{0}^{2}+\Gamma_{a}^{2}\right)}^{4}}\right]$$

Interestingly, there is no linear correction in *T* to *G* in the above formulas.

To end this general theoretical part, and in order to avoid confusion regarding the applicability to real molecular junctions, we want to emphasize that none of the above results quantifying thermal effects on the charge transport via tunneling is limited to a specific experimental platform, be it SET, EGaIn (to be examined in Section 2.3 and Section 2.4), CP-AFM (considered earlier [60]) or any other.

What is important for the single level model underlying Equation (1) is that the charge transport is “one-dimensional”, i.e., proceeds along *individual* molecules; loosely speaking, that an electron (or hole) leaving the left electrode does not tunnel across the left half of a molecule A, then jumps on a neighboring molecule B, and finishes the trip to the right electrode after tunneling across the right half of molecule B.

Importantly, the theoretical single level model utilized does not necessarily rule out an intermolecular (A-B) interaction. In an elementary transport process, an electron tunneling across molecule A can interact with the adjacent molecule B. Provided that the charge transport does not induce electron *exchange* between adjacent molecules A and B, the effects of this potentially significant intermolecular interaction translate into an extra level shift (i.e., renormalized $\varepsilon_{0}$) and an extra level broadening expressed as an additional term to the RHS of Equation (3)

$$\Gamma_{s}+\Gamma_{t}\to \Gamma_{s}+\Gamma_{t}+\Gamma_{env}$$

Above, the subscript “env” stands for environment. Because both $\varepsilon_{0}$ and $\Gamma_{a}$ are model parameters, the implications for data fitting are not dramatic.

The fact that in Section 2.4 we will be able to estimate the fraction *f* of active molecules merely in terms of $\Gamma_{s}$ and $\Gamma_{t}$ (amounting to assume $\Gamma_{env}=0$) demonstrates that, at least for the large area EGaIn-based junctions considered there, intermolecular interaction effects do not have a dramatical impact on transport.

### 2.2. Results Illustrating the Temperature Impact on the Charge Transport by Tunneling

**Figure 3:**
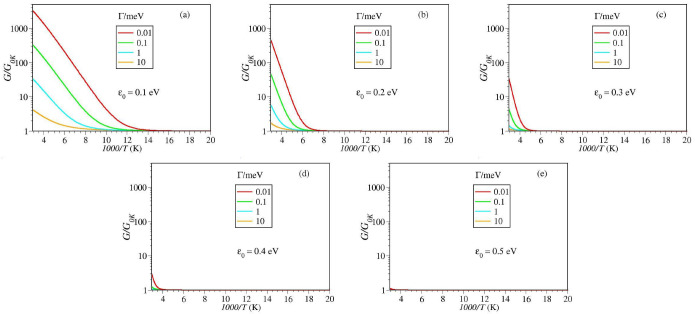
Simulating the temperature impact on the low bias conductance *G* normalized to the zero temperature value $G_{0K}$. Impact of a variable level width offset $\Gamma_{a}$ at fixed energy offset $\varepsilon_{0}$. Notice that the range on the *y*-axis is the same all panels (**a**–**e**). At given $\varepsilon_{0}$, the impact of temperature is more pronounced at smaller $\Gamma_{a}$ As visible, thermal effects on *G* are negligible for $\left|\varepsilon_{0}\right|$ larger than about 0.4 eV.

**Figure 4:**
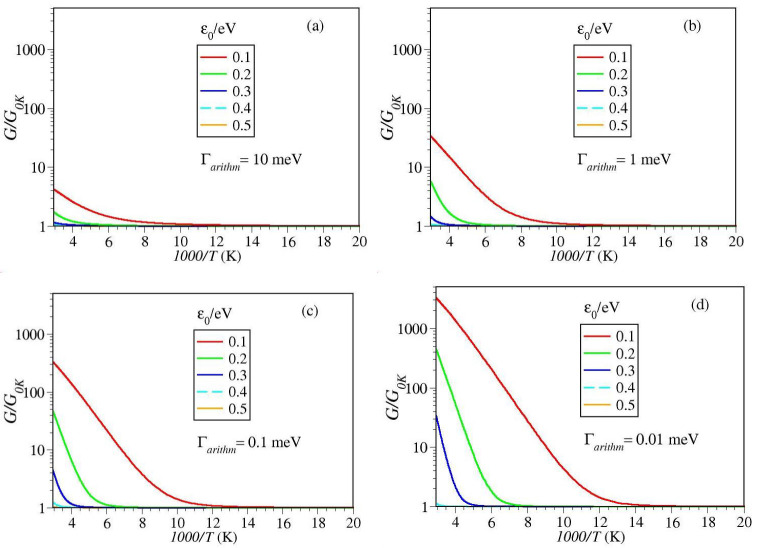
Simulating the temperature impact on the low bias conductance G normalized to the zero temperature value $G_{0K}$ Impact of a variable level offset $\varepsilon_{0}$ at fixed $\Gamma_{a}$ At given $\Gamma_{a}$, the impact of temperature is more pronounced at smaller $\left|\varepsilon_{0}\right|$ Notice that the range on the y-axis is the same all panels (**a**–**d**).

Insight into the thermal impact on the tunneling conductance can be gained by inspecting the results of numerical simulations depicted in Figure 3 and Figure 4. Inspection of these figures reveals that, irrespective of the magnitude of the MO width $\Gamma_{a}$, up to T≈340 K—a value that safely covers the temperature range accessed in experiments [15,60]—thermal effects are negligible for energy offsets $\left|\varepsilon_{0}\right|$ larger than about 0.4 eV (cf. Figure 3d,e).

Below this value, thermal effects become significant. At a given level offset value $\left|\varepsilon_{0}\right|$, they are the more pronounced, the smaller the value of $\Gamma_{a}$ is (cf. Figure 3a–c). Likewise, at given level width $\Gamma_{a}$, thermal effects are the more pronounced, the smaller the level offset $\left|\varepsilon_{0}\right|$ is (cf. Figure 4a–c).

By and large, the message conveyed by Figure 3 and Figure 4 is clear: temperature dependent measured data should by no means be taken as conclusive evidence for two-step hopping conduction (cf. ref. [45] and citations therein).

Figure 3 and Figure 4 clearly illustrate that, for sufficiently (but realistically) small values of $\left|\varepsilon_{0}\right|$ and $\Gamma_{a}$ the single-step tunneling transport can exhibit a strong temperature dependence. At high *T*, the (pseudo-)Arrhenius behavior resulting from tunneling (cf. Equation (9b)) can hardly be distinguished from the traditional Arrhenius characteristic for charge transport via hopping. As the temperature is lowered, this Arrhenius-like regime $G\approx G_{T}$ (cf. Equation (9a)) gradually switches into a Sommerfeld regime [45], wherein thermal effects basically represent corrections (cf. Equation (10)) to the zero temperature value $G_{0K}$ (Equation (8)).

**Figure 5:**
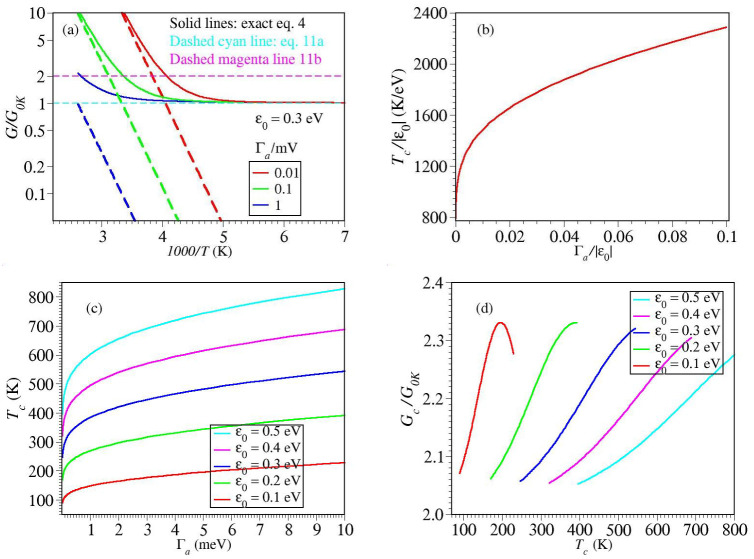
(**a**) In view of the gradual character of the Arrhenius-Sommerfeld transition, a “critical” (“transition”) temperature $T_{c}$ can only be defined by an arbitrary convention, e.g., where the inclined dashed lines depicting $G_{T}$ (Equation (9a)) cross the cyan horizontal line depicting the zero temperature conductance $G_{0K}$. Notice that at $T=T_{c}$ the exact conductance is, roughly, two times larger $G_{0K}$ (horizontal magenta line). (**b**) The curve of the critical temperature in dimensionless coordinates obtained using Equation (11a). (**c**) Curves for $T_{c}$ for various model parameter values indicated in the inset. (**d**) Curves showing that at $T=T_{c}$ the exact conductance $G_{c}=G\left(T_{c}\right)$ is approximately twice the zero temperature conductance $G_{0K}$.

Because this transition is gradual, a crossover (“critical” or “transition”) temperature $T_{c}$ separating these Arrhenius and Sommerfeld regimes can only be defined by some arbitrary convention. An intuitive possibility is to define $T_{c}$ by the point where extrapolated (dashed, nearly linear) curves of $G_{T}$ (Equation (9a)) cross the horizontal (dashed, cyan) line corresponding to the zero temperature value $G_{0K}$, Equation (11a) (Figure 5a). This “critical” temperature $T_{c}$ approximately corresponds to the temperature where the exact, temperature dependent value of *G* represents twice the zero temperature value $G_{0K}$ (Equation (11b), magenta horizontal line in Figure 5a,d).

(11a) $$\begin{array}{ccc} {\left.G_{T}\right|}_{T=Tc} & = & G_{0K} \end{array}$$

(11b) $$\begin{array}{ccc} {\left.G(T)\right|}_{T=Tc} & \approx & 2G_{0K} \end{array}$$

Imposing Equation (11a) yields a curve of $T_{c}$ versus $\Gamma_{a}$ which is unique in the reduced quantities $T_{c}/\left|\varepsilon_{0}\right|$ and $\Gamma_{a}/\left|\varepsilon_{0}\right|$ (cf. Figure 4b). More specific illustrations are depicted in Figure 5c, which give a flavor on the values characterizing real molecular junctions. Noteworthily, the results presented in Figure 5 give additional support to a previous conclusion [50]; contradicting a possible naive expectation, the crossover between a temperature dependent and temperature independent transport by tunneling occurs at a value of $k_{B}T_{c}$ which is, in general, substantially different from $\Gamma_{a}$.

### 2.3. Results for Specific Molecular Junctions

Out of the experimental results available for charge transport through molecular junctions at variable temperature [3,13,15,60,63,64,65,66,67,68,69,70,71], we will consider in this section the junctions fabricated with symmetric molecules consisting of a ferrocene unit (Fc) [72,73] contacted via alkyl spacers to electrodes [15] in two testbeds. In single molecule transistor (SET) setup, −S−(CH_2_)_4_−Fc−(CH_2_)_4_−S^-^ molecules were contacted to gold electrodes via thiol groups. In junctions based on self assembled monolayers (SAM), molecules were sandwiched between gold and EGaIn electrodes (Au−S−(CH_2_)_6_−Fc−(CH_2_)_6_−CH_3_/EGaIn).

We compared the theoretical zero bias conductance G=G(T) with the quantity j(T;V)/V estimated from the experimental currents j(T;V) given in arbitrary units in ref. [15] for the lowest bias *V* (namely, at V=10 mV for SET for 80K≤T≤220 K and at V=160 mV for SAM for 220K≤T≤330 K).

**Figure 6:**
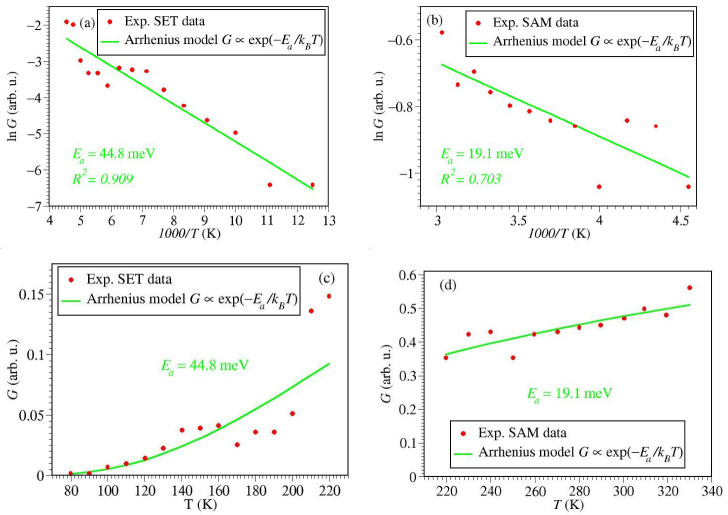
Arrhenius fits (Equation (12)) of the temperature dependent conductance *G* of the ferrocene-based molecular junctions measured in ref. [15] (courtesy of C. A. Nijhuis and Y. Li): (**a**) for SET setup at V=10 mV and (**b**) SAM setup at V=160 mV. They are recast in coordinates *G* versus *T* in (**c**,**d**), respectively. The values of *V* indicated here correspond to the lowest bias results reported [15].

To start with, we present in Figure 6 results obtained by fitting the experimental data (courtesy of C. A. Nijhuis and Y. Li) postulating an Arrhenius dependence

(12) $$G=G_{\infty }\exp\left(-\frac{E_{a}}{k_{B}T}\right)$$

which corresponds to lnG varying linearly with inverse temperature 1/T. The activation energies $E_{a}≃45$ meV for SET and $E_{a}≃160$ meV for SAM obtained using MATHEMATCA’s routine *LinearModelFit* shown in Figure 6 are similar to those from Figure 3 of ref. [15].

However, as seen above, a pure Arrhenius dependence cannot be substantiated by the present model calculations. Model parameters estimated from data fitting using the various methods discussed in Section 2.1 are collected in Table 1. They show that even the pseudo-Arrhenius form $G\to G_{p.A}$ (Equation (9b)), which merely differs from $G_{A}$ by a prefactor ∝1/T, yields significantly different “activation energies” ($\left|\varepsilon_{0}\right|≃56$ meV for SET and $\left|\varepsilon_{0}\right|≃44$ meV for SAM). We put “activation energies” in quotation marks because $\left|\varepsilon_{0}\right|$ does not represent a true barrier energy to be overcome by the charge carriers (in our specific case of HOMO-mediated conduction, holes [15]).

We recast the data depicted in Arrhenius coordinates (lnG versus 1/T, Figure 6a,b) in coordinates *G* versus *T* (Figure 6c,d, respectively) to emphasize that, while not conspicuous for the case of SET, inferring an Arrhenius dependence from the measurements for the SAM-based junctions is highly problematic.

**Figure 7:**
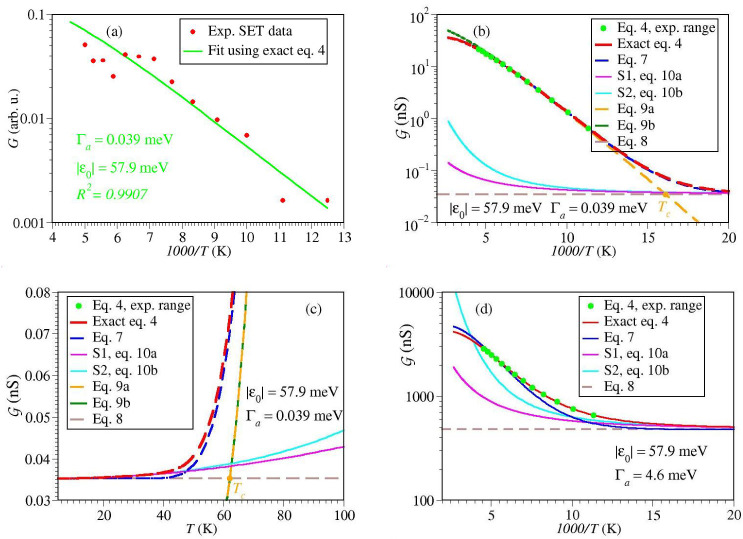
Results for SET setup. (**a**) Conductance (*G*) data at the lowest bias (V=10 mV) reported in ref. [15] fitted using the exact Equation (4). (**b**) Exact curve G versus 1/T extrapolated beyond the temperature range sampled experimentally [15] along with various approximations indicated in the legend. (**c**) Same as (**b**) recast as a function of *T*. (**d**) Same as (**b**) using the width value $\Gamma_{a}$ estimated for the SAM setup. For the meaning of G∝G, see Equation (13a) in the main text.

**Figure 8:**
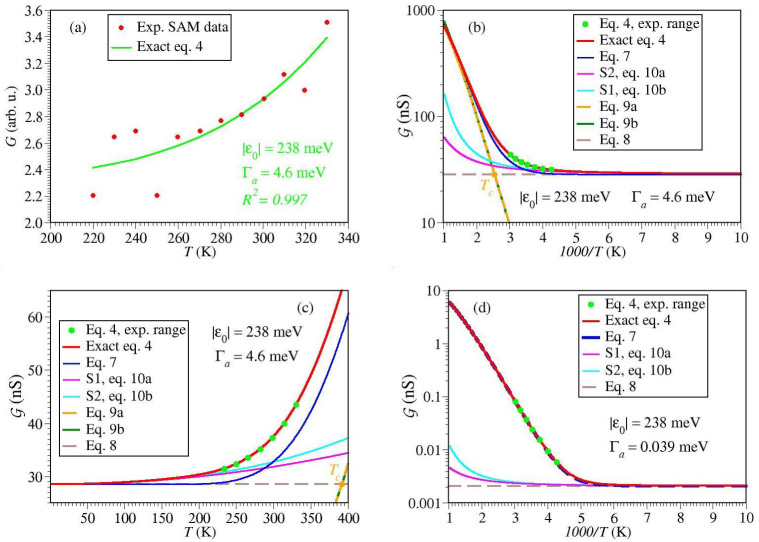
Results for SAM setup. (**a**) Conductance data at the lowest bias (V=160 mV) reported in ref. [15] fitted using the exact Equation (4). (**b**) Exact curve G versus 1/T extrapolated beyond the temperature range sampled experimentally [15] along with various approximations indicated in the legend. (**c**) Same as (**b**) recast as a function of *T*. (**d**) Same as (**b**) using the width value $\Gamma_{a}$ estimated for the SET setup. For the meaning of G∝G, see Equation (13a) in the main text.

Figure 7a and Figure 8a depict data fitting using the exact Equation (4) and MATHEMATICA’s routine *NonlinearModelFit.*Comparison between Figure 6d and Figure 8a makes it clear why the MO energy offset estimated exactly for SAM ($\left|\varepsilon_{0}\right|≃238$ meV) differs by an order of magnitude from the Arrhenius-based activation energy ($E_{a}≃19$ meV). As visible (and also reflected in the different $R^{2}$-values), the fitting curve of Figure 6d better describes the general trend emerging the experimental data than the Arrhenius-based fitting curve of Figure 6d.

This difference is not so pronounced in the SET case (cf. Figure 6a and Figure 7a). This explains why, although significant, the difference between the estimated MO energy offset ($\left|\varepsilon_{0}\right|≃58$ meV) and the Arrhenius-based activation energy ($E_{a}≃45$ meV) is not so dramatically large.

For comparison purposes, along with the exact curves for conductance, in Figure 7b,c and Figure 8b,c we also show curves computed with the same parameters using various approximate formulas presented in Section 2.1. They are depicted for temperature ranges beyond those (indicated by green points) sampled in experiment, in order to emphasize that the experiments of ref. [15] did not sample the Arrhenius-Sommerfeld transition for SET but partially sampled it for SAM.

Figure 7b reveals why for SET experiments Equation (7) represents a much more reasonable approximation than for SAM experiments (Figure 8c). In the former case, the temperatures explored experimentally are well below $T_{c}$ (the value of which is marked by an orange point), while in the latter case they are above $T_{c}$. The small asymptotic (zero temperature) value $G_{0K}$ depicted by the brown dashed line in Figure 7b makes it clear why Equation (9a,b) still reasonably describe the SET experimental data; Equation (9a) reasonably approximates Equation (7) in cases where $G_{0K}$ is small. Again, this is in contrast to the SAM data (Figure 8b,c).

Although the temperatures explored experimentally are above $T_{c}$, thermal effects exhibited by the SAM data do not merely represent corrections to the zero temperature limit. The SAM data do not simply belong to the pure Sommerfeld regime; the magenta (Equation (10a)) and cyan (Equation (10b)) curves in Figure 8b,c do significantly differ from the exact red curve (Equation (4)). In accord to those elaborated in Section 2.1, one could also note here that Figure 8b,c illustrate limitations of the interpolation expressed by Equation (7) in describing the crossover Arrhenius-Sommerfeld regime.

Let us briefly comment on the difference between the parameters for the SET and SAM. The relatively small difference between the values of $\varepsilon_{0}$ extracted form the SET and SAM data (58 meV versus 238 meV, respectively) can reflect effects due to the gate voltage ($V_{g}=-1.5$ V versus $V_{g}=0$) [13,14] and image charges (absent in the former case, present in the latter) [74]. More importantly than differences in $\varepsilon_{0}$, $\Gamma_{a}$’s differ by two orders of magnitude. We assign this difference as an effect of the SAM-driven work function modification δΦ. The strong (exponential) dependence of the molecule-electrode couplings on δΦ was amply documented in earlier studies [32,60,75,76,77].

To emphasize the important role played by $\Gamma_{a}$ in the Arrhenius-Sommerfeld transition, we also show curves for conductance computed for $\varepsilon_{0}$ determined for the SET setup and $\Gamma_{a}$ estimated for the SAM setup (Figure 7d) and vice versa (Figure 8d). In the former case, the temperature range sampled experimentally comprises the crossover region between the Arrhenius and Sommerfeld regimes. In the latter case, the temperature range sampled experimentally is shifted inside the Arrhenius regime.

### 2.4. The Arrhenius-Sommerfeld Thermal Transition: A Possible Approach to Estimate the Number of Molecules in Large Area Tunneling Molecular Junctions

In the various formulas presented above, *G* is the conductance per molecule. Therefore, whatever the method utilized, fitting the transport measurements of ref. [15] encounters an important difficulty: ref. [15] only reported relative currents, not absolute currents. This is why, paradoxically, the discussion of this specific case is significantly more involved than the general methodology (Section 2.5) to be applied in cases where experimentalists report absolute (not relative) values of measured currents.

Fitting relative currents using Equation (9a,b) (as well as Equation (12), which was also used in ref. [15]) merely allows the determination of $\left|\varepsilon_{0}\right|$. Data fitting using Equations (4), (6d,f) or (7) allows to obtain the values of $\left|\varepsilon_{0}\right|$ and $\Gamma_{a}$, but $\Gamma_{g}$ can only be obtained up to an unknown multiplicative factor.

For this reason, the value of $\Gamma_{g}$ was not indicated in Figure 6a and Figure 7a, and *G* was given in arbitrary units. What we showed in Figure 7b–d and Figure 8b–d is the conductance per molecule G defined as

(13a) $$G\equiv {\left.G\right|}_{\Gamma_{g}=\Gamma_{a}}$$

which holds when the MO level is symmetrically coupled to electrodes (cf. Equations (2) and (3))

(13b) $$\Gamma_{a}=\Gamma_{g}\Leftrightarrow \Gamma_{s}=\Gamma_{t}$$

To exemplify this, and for greater clarity, used in conjunction with Equation (4), G is expressed by

(13c) $$\frac{G}{G_{0}}=\frac{\Gamma_{a}}{2\pi k_{B}T}\;Re\;\psi^{\prime }\left(\frac{1}{2}+\frac{\Gamma_{a}}{2\pi k_{B}T}+i\frac{\varepsilon_{0}}{2\pi k_{B}T}\right)$$

The assumption in Equation (13b) is justified for the electrostatically gated SET (Au−S−(CH_2_)_4_−Fc−(CH_2_)_4_−S−Au) symmetrically adsorbed chemically via thiol groups, which are very likely single molecule devices [13,78]. For this reason, G presented in Figure 7b is equal to the true (absolute, i.e., not relative) conductance value *G*. The absolute values calculated in this way appear to be consistent with the absolute values measured in experiment [15], as far as they can be reconstituted after so many years [79].

Obviously, the above approach cannot be applied for the EGaIn large area SAM-based junctions having a nominal (geometric) area of $A_{n}\approx 700\;\mu m^{2}$ [15]. The reason is twofold. First, they comprise an effective number of molecules $N_{eff}>1$. Above, we said “nominal area” and “effective number” because, as well documented [5,6,26,40,41,42,43,44], the effective (“electric”) area $A_{eff}$ may be on orders of magnitude smaller than $A_{n}$, or rephrased, because the total number of molecules $N_{n}$ in the junction is much larger than those effectively involved in charge transport:

(14) $$f=\frac{A_{eff}}{A_{n}}=\frac{N_{eff}}{N_{n}}\ll 1$$

Second, the physical (van der Waals) EGaIn contact with the SAM is quantified by a coupling $\Gamma_{t}\equiv \Gamma_{t}^{EGaIn}$ substantially smaller than the chemical coupling $\Gamma_{s}\equiv \Gamma_{s}^{Au}$ to the gold substrate.

Put together, the following relations relating the presently calculated G and the conductance $G_{j}$ of the measured junction apply

(15a) $$\begin{array}{ccc} G & = & \frac{\Gamma_{t}^{EGaIn}}{\Gamma_{s}^{Au}}G \end{array}$$

(15b) $$\begin{array}{ccc} N_{eff} & = & \frac{G_{j}}{G}=\frac{\Gamma_{s}^{Au}}{\Gamma_{t}^{EGaIn}}\frac{G_{j}}{G} \end{array}$$

(15c) $$\begin{array}{ccc} \Sigma & = & \frac{N_{n}}{A_{n}}=\frac{N_{eff}}{A_{eff}} \end{array}$$

(15d) $$\begin{array}{ccc} \frac{N_{eff}}{N_{n}} & = & \frac{A_{eff}}{A_{n}}=\frac{\Gamma_{s}^{Au}}{\Gamma_{t}^{EGaIn}}\frac{G_{j}}{G}\frac{1}{A_{n}\Sigma } \end{array}$$

Above, Σ stands for the SAM coverage (number of molecules per unit area).

For SAMs of alkyl thiols and oligophenylene thiols utilized to fabricate CP-AFM junctions, measurements via Rutherford backscattering (RBS) and nuclear reaction analysis (NRA) provided coverage values Σ≃3.5 molecules/nm${}^{2}$ practically independent of the type of molecule [80,81].

Experiments have indicated similar coverage values of SAMs anchored via thiols on gold substrate used to fabricate CP-AFM junctions and EGaIn junctions [5]. Therefore, the above value of Σ is also reasonable for the presently considered SAM. For the EGaIn-based junctions of nominal contact area $A_{n}\approx 700\;\mu m^{2}$ of ref. [15], a nominal number of molecules per junction $N_{n}=A_{n}\Sigma \approx 2.45\times 10^{6}$ can thus be estimated.

At room temperature, we obtained the value G≃37 nS. As far as values measured more than eight years ago can be reconstituted [15], a junctions’s conductance $G_{j}\approx 20$ nS can be inferred [82]. For CP-AFM junctions fabricated with alkyl thiols and gold substrate and tip electrodes, we recently estimated a ratio between the thiol chemisorbed contact and the methyl physisorbed contact of

(16) $$\frac{\Gamma_{s}^{Au}}{\Gamma_{t}^{Au}}≃37$$

If we used these values, we would deduce from Equation (15a) a value $N_{eff}\approx 20$, amounting to $f=N_{eff}/N_{n}=A_{eff}/A_{n}\approx 10^{-5}$. However, for the reason explained below, this value is underestimated.

Equation (16) assumed that both (substrate and tip/top) electrodes are of gold, which does not apply to the presently considered Au-(…Fc…)/EGaIn junctions. The EGaIn top electrode has a significantly different work function from gold. Using the dependence on the work function Φ of the effective coupling for CP-AFM junctions fabricated with alkyl monothiols (label *m*) and alkyl dithiols (label *d*) [77], we deduced

(17) $$\Gamma_{m,d}\propto e^{\delta_{d,m}\;\Phi }$$

where $\delta_{m}=1.377\;{eV}^{-1}$ and $\delta_{d}=0.998\;{eV}^{-1}$. Following the method presented in ref. [51], we get

(18) $$\Gamma_{t}\propto e^{\left(2\delta_{m}-\delta_{d}\right)\;\Phi }$$

and this yields

(19) $$\frac{\Gamma_{t}^{Au}}{\Gamma_{t}^{EGaIn}}\approx \exp\left[\left(2\delta_{m}-\delta_{d}\right)\left(\Phi_{Au}-\Phi_{EGaIn}\right)\right]\approx 7$$

Above, we used the values $\Phi_{EGaIn}=4.1$ eV and $\Phi_{Au}=5.2$ eV. The fact that $G\propto \Gamma_{g}^{2}\propto \Gamma_{t}$ (cf. Equation (2)) translates into a corrected value

(20) $$\frac{\Gamma_{s}^{\mathrm{Au}}}{\Gamma_{t}^{EGaIn}}=\frac{\Gamma_{t}^{\mathrm{Au}}}{\Gamma_{t}^{EGaIn}}\times \frac{\Gamma_{s}^{\mathrm{Au}}}{\Gamma_{t}^{\mathrm{Au}}}\approx 260$$

to be used instead of Equation (16) to compute *f*. With the above value, Equation (15b,c) yield $N_{eff}\approx 140$ and $A_{eff}\approx 40\;{nm}^{2}$. Indeed, these values are substantially smaller than $N_{n}\approx 2.45\times 10^{6}$ and $A_{n}\approx 700\;\mu m^{2}$ indicated above. This amounts to

(21) $$f=N_{eff}/N_{n}=A_{eff}/A_{n}\approx 0.6\times 10^{-4}$$

This fraction is comparable with area correction factors obtained using completely different methods reported earlier [27] for other EGaIn-based junctions. Possibly, this value is a general characteristics of the platforms using EGaIn top electrodes.

We have also to mention that oligophenyleneimines (OPI) junctions fabricated using EGaIn/Au electrodes were claimed [5] to be 100 times more resistive than OPI Au/Au CP-AFM junctions. The foregoing analysis found that Fc-based EGaIn junctions with alkyl thiol spacers are (only) seven times (cf. Equation (19)) more resistive than similar CP-AFM junctions. This suggests that care should be taken when comparing conducting properties of EGaIn and CP-AFM junctions fabricated with different molecular species, e.g., one should distinguish between localized electrons contributing to the dominant (HO)MO (read Fc-based junctions of ref. [15]) and delocalized electrons (read OPI-based junctions of ref. [5]).

### 2.5. Workflow for Data Fitting

**Figure 9:**
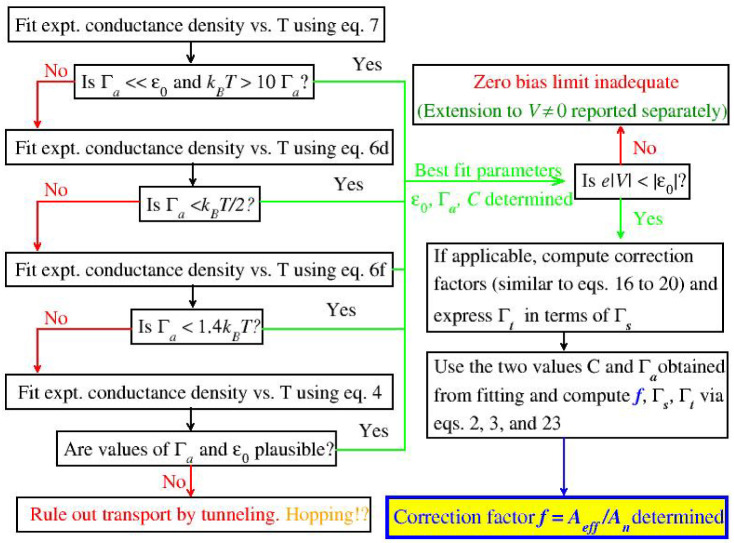
Diagram depicting the workflow for the presently proposed data fitting approach.

In the attempt to aid experimentalists in extracting information from low bias conductance measured at variable temperature, the workflow for the presently proposed data fitting is summarized in the diagram depicted in Figure 9. In addition, a few more details may be useful.

Experiments for large area junctions typically report current densities $j_{exp}\equiv j_{n}$, more precisely, current (*I*) values divided by the junction’s *nominal* area $A_{n}$. In the present low bias framework, the envisaged experimental quantity is the nominal conductance density $g_{exp}\left(T\right)\equiv g_{n}\left(T\right)$. Straightforward manipulation yields

(22) $$g_{n}\left(T\right)\equiv {\left.\frac{j_{n}\left(V;T\right)}{V}\right|}_{V\approx 0}\equiv {\left.\frac{1}{A_{n}}\frac{I(V;T)}{V}\right|}_{V\approx 0}\equiv \frac{G_{j}\left(T\right)}{A_{n}}=\frac{A_{eff}}{A_{n}}\frac{N_{eff}}{A_{eff}}G\left(T\right)=\left(f\Sigma \right)G\left(T\right)$$

where Σ is the SAM coverage. one should note that, whether data fitting is based on the exact Equation (4) or the various approximations based on it—namely, Equations (7), (6d) or (6f)—, the quantity $\Gamma_{g}^{2}$ always enters as a multiplicative factor the RHS of all those expression. Therefore, by stroke of Equation (22), one can use the combination

(23) $$C\equiv f\Sigma \Gamma_{g}^{2}$$

as a unique fitting parameter. Data fitting based on any of the formulas mentioned above yields best fit estimates for $\varepsilon_{0}$, $\Gamma_{a}$, and *C*. The area correction factor *f* can be estimated from *C* and $\Gamma_{a}$ by stroke of Equations (2), (3) and (23) via the additional quantities $\Gamma_{s}$ and $\Gamma_{t}$, provided that an additional relationship between $\Gamma_{s}$ and $\Gamma_{t}$ exists.

For a specific illustration of how this relationship can be obtained for EGaIn-based large area junctions with alkyl spacers, see Equations (16)–(20). A similar strategy can be adopted in case of molecules of oligophenyls [51,76] and oligoacenes [32], for which the contact conductance data ($G_{C}\propto \Gamma_{g}^{2}=\Gamma_{s}\Gamma_{t}$) are also available.

The EGaIn-based junctions represent perhaps the most difficult case to handle. For other platforms (e.g., CP-AFM or crossed-wire [21,22,24]) using symmetric molecules symmetrically contacted to electrodes, the values of $\Gamma_{g}=\Gamma_{a}=\Gamma_{s}=\Gamma_{t}$ and *f* can be straightforwardly be estimated from Equation (23). Obviously, provided that absolute values of the current (conductance) are available, there is no problem at all in the case single molecule junctions. There, $N_{eff}=N_{n}=1$, and $\Gamma_{g,a}$ and $\varepsilon_{0}$ can be directly obtained from data fitting.

## 3. Method

The method utilized in ths paper is based on the general Keldysh formalism [46,47,48,49] applied to the specified molecular junctions considered.

## 4. Conclusions

Routinely, a curve in “Arrhenius” plane (lnG versus 1/T) which is a straight line is taken as evidence for charge transport via a two-step hopping mechanism, while a plot switching from a linear, inclined line to a horizontal line is taken as revealing a transition from two-step hopping to single-step tunneling conduction [83,84], and a curve having a slope of magnitude progressively decreasing as 1/T increases is claimed to indicate a variable range hopping mechanism [85].

The curves presented in this paper (e.g., Figure 3 and Figure 4) demonstrate the drastic limitation of the oversimplified view delineated above. As we showed, all the aforementioned dependencies are fully compatible with a single-step coherent tunneling conduction. In a sufficiently broad temperature range, any curve *G* versus 1/T computed by assuming a single-step tunneling mechanism switches from a roughly exponential shape (Arrhenius-like regime) at high *T* to a less and less *T*-dependent Sommerfeld regime [45] at low *T*.

Whether only one of these regimes or both of them can be accessed in a real molecular junction depends, e.g., on the value of the crossover (“critical”) temperature $T_{c}$ (cf. Section 2.2 and Figure 5), on the temperature range that can be sampled experimentally, or on the thermal stability of the active molecule or electrodes. The latter are significant, e.g., for protein-based and EGaIn-based junctions, which can be employed in a rather restricted temperature range.

The Arrhenius-Sommerfeld transition can be more or less gradual. This is basically controlled by two parameters (level broadening $\Gamma_{a}$ and $\varepsilon_{0}$), which also set the value of $T_{c}$. Unlike $\varepsilon_{0}$ and $\Gamma_{a}$, $\Gamma_{g}$ essentially determines the magnitude of *G*; $T_{c}$ does not depend on $\Gamma_{g}$. In situations far apart from symmetry (e.g., $\Gamma_{s}\ll \Gamma_{t}\to \Gamma_{g}\ll \Gamma_{a}$), $\Gamma_{g}$ only indirectly affects the aforementioned interplay, in the sense that, if *G* is too small at some temperatures, the corresponding *T*-range is experimentally irrelevant.

The various theoretical formulas, expressed in closed analytic forms, reported in this paper aim at assisting experimentalists in processing transport data measured at variable temperatures.

As an important application of those formulas, we used experimental data for ferrocene-based molecular junctions with an EGaIn top electrode to illustrate the possibility of estimating the number of molecules per junction, which is a property of paramount importance for large area junctions, wherein the effective (“electric”) area $A_{eff}$ can and does drastically differ from the nominal (“geometric”) area $A_{n}$. For the specific junctions considered, we obtained a value $A_{eff}/A_{n}\approx 0.6\times 10^{-4}$ compatible with other estimates for EGaIn-based junctions [27]. To facilitate understanding practical details in implementing the presently proposed method of estimating the ratio $A_{\mathrm{eff}}/A_{n}$, we showed a workflow diagram in Figure 9.

In this context, the advantage of the present formulas for G=G(T)—Equations (4), (6d,f) and (7)—as compared to other Arrhenius flavors (Equations (9a,b) and (12)) used previously in the literature becomes more evident. What the latter formulas can provide is merely an “activation energy” whose physical content is more or less obscure. In those formulas, $N\Gamma_{g}^{2}\to N_{eff}\Gamma_{g}^{2}$ enters as a unique fitting parameter. From the best fit estimate, $\Gamma_{g}$ can be computed only in situations where the effective number of molecules per junction $N_{\mathrm{eff}}$ is known, but it is just this quantity that is the most problematic in case of large area junctions. For a similar reason, $N_{\mathrm{eff}}/N_{n}$ cannot be confidently determined for cases where the experimentally accessed *T*-range merely lies in the nearly exponential fall-off (Arrhenius-like) part of the *G*-curve; the very weak dependence of *G* on $\Gamma_{a}$ in such situations makes even the estimate for $\Gamma_{a}$ unreliable.

Fortunately, this was not an impediment in the case of SET [15] examined in Section 2.3; although all measured data belong to the Arrhenius regime, the common value of $\Gamma_{a}\approx \Gamma_{g}$ can be estimated for symmetric, single-molecule platforms. With regard to the other (SAM-based) platform considered, the complete characterization of the SAM-based Fc junctions presented in Section 2.3 was possible just because the temperature range explore experimentally overlaps the Arrhenius-Sommerfeld crossover regime.

To end, we note that the determination of the number of molecules is an important issue not only for large area junctions but also, e.g., for CF-AFM junctions. Although models of contact mechanics [86,87,88] can be very useful to estimate the number of molecules in CP-AFM junctions [76,77,81], reliable information needed (e.g., values of SAM’s Young moduli [89]) is often missing. The present method to estimate $N_{eff}$ can also applied for the CP-AFM platform.

Finally, we emphasize that the entire analysis elaborated in the present paper refers to the transport by tunneling; a coherent, single-step mechanism wherein (say,) electron (or hole) transfer from the left electrode to the (active) molecule is a process that cannot be separated from the electron transfer from the molecule to the right electrode. We did not consider the interplay between transport via tunneling and transport via hopping, which is a two-step mechanism wherein electron transfer from the left electrode to the molecule and electron transfer from the molecule to the right electrode are two distinct, uncorrelated processes characterized by durations much shorter than the electron’s residence time on the molecule, which is sufficiently long to allow molecular reorganization [90]. A possible protocol to disentangle between tunneling and hopping conduction has been proposed [50] and applied [60] elsewhere.

## Figures and Tables

**Table 1:** Parameter values estimated using various methods discussed in the main text. All quantities in meV.

Method	Property	SET	SAM
Equation (12)	$E_{a}$	45	19
Equation (9b)	$\left|\varepsilon_{0}\right|$	56	44
Equation (9a)	$\left|\varepsilon_{0}\right|$	57	53
Equation (7)	$\left|\varepsilon_{0}\right|$	58	193
	$\Gamma_{a}$	0.046	11
Equations (4), (6d) or (6f)	$\left|\varepsilon_{0}\right|$	58	238
	$\Gamma_{a}$	0.039	4.6

## Data Availability

Reasonable data request should be addressed to the author.

## References

[1] Salomon A., Cahen D., Lindsay S., Tomfohr J., Engelkes V., Frisbie C. (2003). Comparison of Electronic Transport Measurements on Organic Molecules. Adv. Mater..

[2] McCreery R.L., Bergren A.J. (2009). Progress with Molecular Electronic Junctions: Meeting Experimental Challenges in Design and Fabrication. Adv. Mater..

[3] McCreery R.L., Yan H., Bergren A.J. (2013). A critical perspective on molecular electronic junctions: There is plenty of room in the middle. Phys. Chem. Chem. Phys..

[4] Xiang D., Wang X., Jia C., Lee T., Guo X. (2016). Molecular-Scale Electronics: From Concept to Function. Chem. Rev..

[5] Sangeeth C.S.S., Demissie A.T., Yuan L., Wang T., Frisbie C.D., Nijhuis C.A. (2016). Comparison of DC and AC Transport in 1.5–7.5 nm Oligophenylene Imine Molecular Wires across Two Junction Platforms: Eutectic Ga-In versus Conducting Probe Atomic Force Microscope Junctions. J. Am. Chem. Soc..

[6] Mukhopadhyay S., Karuppannan S.K., Guo C., Fereiro J.A., Bergren A., Mukundan V., Qiu X., Castaneda Ocampo O.E., Chen X., Chiechi R.C. (2020). Solid-State Protein Junctions: Cross-Laboratory Study Shows Preservation of Mechanism at Varying Electronic Coupling. iScience.

[7] Reed M.A., Zhou C., Muller C.J., Burgin T.P., Tour J.M. (1997). Conductance of a Molecular Junction. Science.

[8] Lörtscher E., Weber H.B., Riel H. (2007). Statistical Approach to Investigating Transport through Single Molecules. Phys. Rev. Lett..

[9] Reichert J., Ochs R., Beckmann D., Weber H.B., Mayor M., Löhneysen H.V. (2002). Driving Current through Single Organic Molecules. Phys. Rev. Lett..

[10] Xu B., Tao N.J. (2003). Measurement of Single-Molecule Resistance by Repeated Formation of Molecular Junctions. Science.

[11] Venkataraman L., Klare J.E., Nuckolls C., Hybertsen M.S., Steigerwald M.L. (2006). Dependence of Single-Molecule Junction Conductance on Molecular Conformation. Nature.

[12] Tal O., Krieger M., Leerink B., van Ruitenbeek J.M. (2008). Electron-Vibration Interaction in Single-Molecule Junctions: From Contact to Tunneling Regimes. Phys. Rev. Lett..

[13] Song H., Kim Y., Jang Y.H., Jeong H., Reed M.A., Lee T. (2009). Observation of Molecular Orbital Gating. Nature.

[14] Song H., Reed M.A., Lee T. (2011). Single Molecule Electronic Devices. Adv. Mater..

[15] Garrigues A.R., Yuan L., Wang L., Singh S., del Barco E., Nijhuis C.A. (2016). Temperature Dependent Charge Transport across Tunnel Junctions of Single-Molecules and Self-Assembled Monolayers: A Comparative Study. Dalton Trans..

[16] Wold D.J., Frisbie C.D. (2000). Formation of Metal-Molecule-Metal Tunnel Junctions: Microcontacts to Alkanethiol Monolayers with a Conducting AFM Tip. J. Am. Chem. Soc..

[17] Wold D.J., Frisbie C.D. (2001). Fabrication and Characterization of Metal-Molecule-Metal Junctions by Conducting Probe Atomic Force Microscopy. J. Am. Chem. Soc..

[18] Beebe J.M., Engelkes V.B., Miller L.L., Frisbie C.D. (2002). Contact Resistance in Metal-Molecule-Metal Junctions Based on Aliphatic SAMs: Effects of Surface Linker and Metal Work Function. J. Am. Chem. Soc..

[19] Wold D.J., Haag R., Rampi M.A., Frisbie C.D. (2002). Distance Dependence of Electron Tunneling through Self-Assembled Monolayers Measured by Conducting Probe Atomic Force Microscopy: Unsaturated versus Saturated Molecular Junctions. J. Phys. Chem. B.

[20] Engelkes V.B., Beebe J.M., Frisbie C.D. (2004). Length-Dependent Transport in Molecular Junctions Based on SAMs of Alkanethiols and Alkanedithiols: Effect of Metal Work Function and Applied Bias on Tunneling Efficiency and Contact Resistance. J. Am. Chem. Soc..

[21] Kushmerick J.G., Holt D.B., Pollack S.K., Ratner M.A., Yang J.C., Schull T.L., Naciri J., Moore M.H., Shashidhar R. (2002). Effect of Bond-Length Alternation in Molecular Wires. J. Am. Chem. Soc..

[22] Kushmerick J.G., Holt D.B., Yang J.C., Naciri J., Moore M.H., Shashidhar R. (2002). Metal-Molecule Contacts and Charge Transport across Monomolecular Layers: Measurement and Theory. Phys. Rev. Lett..

[23] Kushmerick J.G. (2005). Metal-molecule contacts. Mater. Today.

[24] Beebe J.M., Kim B., Gadzuk J.W., Frisbie C.D., Kushmerick J.G. (2006). Transition from Direct Tunneling to Field Emission in Metal-Molecule-Metal Junctions. Phys. Rev. Lett..

[25] Beebe J.M., Kim B., Frisbie C.D., Kushmerick J.G. (2008). Measuring Relative Barrier Heights in Molecular Electronic Junctions with Transition Voltage Spectroscopy. ACS Nano.

[26] Simeone F.C., Yoon H.J., Thuo M.M., Barber J.R., Smith B., Whitesides G.M. (2013). Defining the Value of Injection Current and Effective Electrical Contact Area for EGaIn-Based Molecular Tunneling Junctions. J. Am. Chem. Soc..

[27] Yoon H.J., Bowers C.M., Baghbanzadeh M., Whitesides G.M. (2014). The Rate of Charge Tunneling Is Insensitive to Polar Terminal Groups in Self-Assembled Monolayers in AgTSS(CH_2_)nM(CH_2_)mT//Ga_2_O_3_/EGaIn Junctions. J. Am. Chem. Soc..

[28] Zhao Z., Soni S., Lee T., Nijhuis C.A., Xiang D. (2022). Smart Eutectic Gallium-Indium: From Properties to Applications. Adv. Mater..

[29] Park S., Kang S., Yoon H.J. (2019). Power Factor of One Molecule Thick Films and Length Dependence. ACS Cent. Sci..

[30] Guo S., Hihath J., Diez-Pérez I., Tao N. (2011). Measurement and Statistical Analysis of Single-Molecule Current-Voltage Characteristics, Transition Voltage Spectroscopy, and Tunneling Barrier Height. J. Am. Chem. Soc..

[31] Li C., Pobelov I., Wandlowski T., Bagrets A., Arnold A., Evers F. (2008). Charge Transport in Single Au|Alkanedithiol|Au Junctions: Coordination Geometries and Conformational Degrees of Freedom. J. Am. Chem. Soc..

[32] Kim B., Choi S.H., Zhu X.Y., Frisbie C.D. (2011). Molecular Tunnel Junctions Based on *π*-Conjugated Oligoacene Thiols and Dithiols between Ag, Au, and Pt Contacts: Effect of Surface Linking Group and Metal Work Function. J. Am. Chem. Soc..

[33] Thuo M.M., Reus W.F., Nijhuis C.A., Barber J.R., Kim C., Schulz M.D., Whitesides G.M. (2011). Odd-Even Effects in Charge Transport across Self-Assembled Monolayers. J. Am. Chem. Soc..

[34] Ramin L., Jabbarzadeh A. (2011). Odd–Even Effects on the Structure, Stability, and Phase Transition of Alkanethiol Self-Assembled Monolayers. Langmuir.

[35] Baghbanzadeh M., Simeone F.C., Bowers C.M., Liao K.C., Thuo M., Baghbanzadeh M., Miller M.S., Carmichael T.B., Whitesides G.M. (2014). Odd-Even Effects in Charge Transport across n-Alkanethiolate-Based SAMs. J. Am. Chem. Soc..

[36] Jiang L., Sangeeth C.S.S., Nijhuis C.A. (2015). The Origin of the Odd-Even Effect in the Tunneling Rates across EGaIn Junctions with Self-Assembled Monolayers (SAMs) of n-Alkanethiolates. J. Am. Chem. Soc..

[37] Nurbawono A., Liu S., Nijhuis C.A., Zhang C. (2015). Odd-Even Effects in Charge Transport through Self-Assembled Monolayer of Alkanethiolates. J. Phys. Chem. C.

[38] Song P., Thompson D., Annadata H.V., Guerin S., Loh K.P., Nijhuis C.A. (2017). Supramolecular Structure of the Monolayer Triggers Odd-Even Effects in the Tunneling Rates across Noncovalent Junctions on Graphene. J. Phys. Chem. C.

[39] Ben Amara F., Dionne E.R., Kassir S., Pellerin C., Badia A. (2020). Molecular Origin of the Odd-Even Effect of Macroscopic Properties of n-Alkanethiolate Self-Assembled Monolayers: Bulk or Interface?. J. Am. Chem. Soc..

[40] Selzer Y., Cai L., Cabassi M.A., Yao Y., Tour J.M., Mayer T.S., Allara D.L. (2005). Effect of Local Environment on Molecular Conduction: Isolated Molecule versus Self-Assembled Monolayer. Nano Lett..

[41] Milani F., Grave C., Ferri V., Samori P., Rampi M.A. (2007). Ultrathin *π*-Conjugated Polymer Films for Simple Fabrication of Large-Area Molecular Junctions. ChemPhysChem.

[42] Akkerman H.B., de Boer B. (2008). Electrical Conduction through Single Molecules and Self-Assembled Monolayers. J. Phys. Condens. Matt..

[43] Suchand Sangeeth C.S., Wan A., Nijhuis C.A. (2015). Probing the nature and resistance of the molecule-electrode contact in SAM-based junctions. Nanoscale.

[44] Vilan A., Aswal D., Cahen D. (2017). Large-Area, Ensemble Molecular Electronics: Motivation and Challenges. Chem. Rev..

[45] Bâldea I. (2022). Exact Analytic Formula for Conductance Predicting a Tunable Sommerfeld-Arrhenius Thermal Transition within a Single-Step Tunneling Mechanism in Molecular Junctions Subject to Mechanical Stretching. Adv. Theor. Simul..

[46] Caroli C., Combescot R., Nozieres P., Saint-James D. (1971). Direct Calculation of the Tunneling Current. J. Phys. C Solid State Phys..

[47] Meir Y., Wingreen N.S. (1992). Landauer formula for the current through an interacting electron region. Phys. Rev. Lett..

[48] Haug H.J.W., Jauho A.P. (2008). Quantum Kinetics in Transport and Optics of Semiconductors.

[49] Cuevas J.C., Scheer E. (2017). Molecular Electronics: An Introduction to Theory and Experiment.

[50] Bâldea I. (2017). Protocol for Disentangling the Thermally Activated Contribution to the Tunneling-Assisted Charge Transport. Analytical Results and Experimental Relevance. Phys. Chem. Chem. Phys..

[51] Bâldea I. (2021). Why asymmetric molecular coupling to electrodes cannot be at work in real molecular rectifiers. Phys. Rev. B.

[52] Sommerfeld A., Bethe H., Scheel G. (1933). Elektronentheorie der Metalle. Handbuch der Physik.

[53] Desjonqueres M.C., Spanjaard D. (1996). Concepts in Surface Physics.

[54] Neaton J.B., Hybertsen M.S., Louie S.G. (2006). Renormalization of Molecular Electronic Levels at Metal-Molecule Interfaces. Phys. Rev. Lett..

[55] Bâldea I. (2014). Single-Molecule Junctions Based on Bipyridine: Impact of an Unusual Reorganization on the Charge Transport. J. Phys. Chem. C.

[56] Bâldea I. (2014). Quantifying the Relative Molecular Orbital Alignment for Molecular Junctions with Similar Chemical Linkage to Electrodes. Nanotechnology.

[57] Abramowitz M., Stegun I.A. (1964). Handbook of Mathematical Functions with Formulas, Graphs, and Mathematical Tables.

[58] Bâldea I. (2012). Interpretation of Stochastic Events in Single-Molecule Measurements of Conductance and Transition Voltage Spectroscopy. J. Am. Chem. Soc..

[59] Sedghi G., Garcia-Suarez V.M., Esdaile L.J., Anderson H.L., Lambert C.J., Martin S., Bethell D., Higgins S.J., Elliott M., Bennett N. (2011). Long-Range Electron Tunnelling in Oligo-Porphyrin Molecular Wires. Nat. Nanotechnol..

[60] Smith C.E., Xie Z., Bâldea I., Frisbie C.D. (2018). Work Function and Temperature Dependence of Electron Tunneling through an N-Type Perylene Diimide Molecular Junction with Isocyanide Surface Linkers. Nanoscale.

[61] Jahnke E., Emde F. (1945). Tables of Functions with Formulae and Curves.

[62] Ashcroft N.W., Mermin N.D. (1976). Solid State Physics.

[63] Poot M., Osorio E., O’Neill K., Thijssen J.M., Vanmaekelbergh D., van Walree C.A., Jenneskens L.W., van der Zant H.S.J. (2006). Temperature Dependence of Three-Terminal Molecular Junctions with Sulfur End-Functionalized Tercyclohexylidenes. Nano Lett..

[64] Heimbuch R., Wu H., Kumar A., Poelsema B., Schön P., Vancso G.J., Zandvliet H.J.W. (2012). Variable-Temperature Study of the Transport Through a Single Octanethiol Molecule. Phys. Rev. B.

[65] Asadi K., Kronemeijer A.J., Cramer T., Jan Anton Koster L., Blom P.W.M., de Leeuw D.M. (2013). Polaron hopping mediated by nuclear tunnelling in semiconducting polymers at high carrier density. Nat. Commun..

[66] Xiang L., Hines T., Palma J.L., Lu X., Mujica V., Ratner M.A., Zhou G., Tao N. (2016). Non-Exponential Length Dependence of Conductance in Iodide-Terminated Oligothiophene Single-Molecule Tunneling Junctions. J. Am. Chem. Soc..

[67] McCreery R.L. (2016). Effects of Electronic Coupling and Electrostatic Potential on Charge Transport in Carbon-Based Molecular Electronic Junctions. Beilstein J. Nanotechnol..

[68] Kumar K.S., Pasula R.R., Lim S., Nijhuis C.A. (2016). Long-Range Tunneling Processes across Ferritin-Based Junctions. Adv. Mater..

[69] Xin N., Jia C., Wang J., Wang S., Li M., Gong Y., Zhang G., Zhu D., Guo X. (2017). Thermally Activated Tunneling Transition in a Photoswitchable Single-Molecule Electrical Junction. J. Phys. Chem. Lett..

[70] Morteza Najarian A., McCreery R.L. (2017). Structure Controlled Long-Range Sequential Tunneling in Carbon-Based Molecular Junctions. ACS Nano.

[71] Xin N., Hu C., Al Sabea H., Zhang M., Zhou C., Meng L., Jia C., Gong Y., Li Y., Ke G. (2021). Tunable Symmetry-Breaking-Induced Dual Functions in Stable and Photoswitched Single-Molecule Junctions. J. Am. Chem. Soc..

[72] Haaland A., Nilsson J.E. (1968). The Determination of Barriers to Internal Rotation by Means of Electron Diffraction. Ferrocene and Ruthenocene. Acta Chem. Scand..

[73] Coriani S., Haaland A., Helgaker T., Jorgensen P. (2006). The Equilibrium Structure of Ferrocene. ChemPhysChem.

[74] Bâldea I. (2013). Transition Voltage Spectroscopy Reveals Significant Solvent Effects on Molecular Transport and Settles an Important Issue in Bipyridine-Based Junctions. Nanoscale.

[75] Xie Z., Bâldea I., Smith C., Wu Y., Frisbie C.D. (2015). Experimental and Theoretical Analysis of Nanotransport in Oligophenylene Dithiol Junctions as a Function of Molecular Length and Contact Work Function. ACS Nano.

[76] Xie Z., Bâldea I., Frisbie C.D. (2019). Determination of Energy Level Alignment in Molecular Tunnel Junctions by Transport and Spectroscopy: Self-Consistency for the Case of Oligophenylene Thiols and Dithiols on Ag, Au, and Pt Electrodes. J. Am. Chem. Soc..

[77] Xie Z., Bâldea I., Frisbie C.D. (2019). Energy Level Alignment in Molecular Tunnel Junctions by Transport and Spectroscopy: Self-Consistency for the Case of Alkyl Thiols and Dithiols on Ag, Au, and Pt Electrodes. J. Am. Chem. Soc..

[78] Bâldea I., Köppel H. (2012). Evidence on single-molecule transport in electrostatically-gated molecular transistors. Phys. Lett. A.

[79] del Barco E. Private commuication (University of Central Florida, 2022).

[80] Demissie A.T., Haugstad G., Frisbie C.D. (2016). Quantitative Surface Coverage Measurements of Self-Assembled Monolayers by Nuclear Reaction Analysis of Carbon-12. J. Phys. Chem. Lett..

[81] Xie Z., Bâldea I., Demissie A.T., Smith C.E., Wu Y., Haugstad G., Frisbie C.D. (2017). Exceptionally Small Statistical Variations in the Transport Properties of Metal-Molecule-Metal Junctions Composed of 80 Oligophenylene Dithiol Molecules. J. Am. Chem. Soc..

[82] Li Y. Private commuication (Tsingua University, 2022).

[83] Choi S.H., Kim B., Frisbie C.D. (2008). Electrical Resistance of Long Conjugated Molecular Wires. Science.

[84] Hines T., Diez-Perez I., Hihath J., Liu H., Wang Z.S., Zhao J., Zhou G., Müllen K., Tao N. (2010). Transition from Tunneling to Hopping in Single Molecular Junctions by Measuring Length and Temperature Dependence. J. Am. Chem. Soc..

[85] Shklovskii B.I., Efros A.L. (1984). Variable-Range Hopping Conduction. Electronic Properties of Doped Semiconductors.

[86] Maugis D. (1992). Adhesion of Spheres: The JKR-DMT Transition Using a Dugdale Model. J. Colloid. Interf. Sci..

[87] Johnson K.L. (1985). Contact Mechanics.

[88] Haugstad G. (2012). Atomic Force Microscopy.

[89] Bâldea I. (2021). Self-assembled monolayers of oligophenylenes stiffer than steel and silicon, possibly even stiffer than Si3N4. Appl. Surf. Sci. Adv..

[90] Bâldea I. (2013). Important Insight into Electron Transfer in Single-Molecule Junctions Based on Redox Metalloproteins from Transition Voltage Spectroscopy. J. Phys. Chem. C.

